# Bulky Pulmonary Mucosa-Associated Lymphoid Tissue Lymphoma Treated with Yttrium-90 Ibritumomab Tiuxetan

**DOI:** 10.1155/2013/675187

**Published:** 2013-11-26

**Authors:** Shinobu Tamura, Tokuji Ikeda, Toshio Kurihara, Yoshiteru Kakuno, Hideki Nasu, Yoshio Nakano, Koichi Oshima, Tokuzo Fujimoto

**Affiliations:** ^1^Department of Internal Medicine, Social Insurance Kinan Hospital, 46-70 Shinjo, Tanabe, Wakayama 646-8588, Japan; ^2^Department of Pharmacy, Social Insurance Kinan Hospital, 46-70 Shinjo, Tanabe, Wakayama 646-8588, Japan; ^3^Department of Radiology, Social Insurance Kinan Hospital, 46-70 Shinjo, Tanabe, Wakayama 646-8588, Japan; ^4^Department of Pathology, School of Medicine, Kurume University, 67 Asahimati, Kurume 830-0011, Japan

## Abstract

An 84-year-old woman was admitted to our hospital with nonproductive cough and dyspnea on exertion. Computed tomography (CT) scan revealed extensive consolidation in the right lung. She was diagnosed with pulmonary mucosa-associated lymphoid tissue (MALT) lymphoma using CT-guided lung biopsy. Her pulmonary images and respiratory symptoms did not improve two months after receiving 4 cycles of rituximab weekly; therefore, yttrium-90 ibritumomab tiuxetan was chosen as salvage therapy. The abnormal shadow on her pulmonary images was significantly reduced two months later, and she had no symptoms without nonhematological toxicities. She has had no progression for 18 months. Furthermore, radiation pneumonitis has not also been observed. We herein reported bulky pulmonary MALT lymphoma treated with yttrium-90 ibritumomab tiuxetan.

## 1. Introduction

Mucosa-associated lymphoid tissue (MALT) lymphoma has been shown to develop in various mucosal tissues, such as the gastrointestinal tract, salivary glands, thyroid gland, and orbital cavity [1, 2]. Primary pulmonary malignant lymphomas are relatively rare, with the majority being of the MALT type [3–5]. Pulmonary MALT lymphoma also originates in bronchial mucosa-associated lymphoid tissue. A definitive diagnosis using noninvasive techniques including transbronchial lung biopsy (TBLB) and/or computed tomography (CT) guided lung biopsy is difficult in many cases [4, 5]. Therefore, an invasive procedure such as thoracoscopy/open chest pneumonectomy is required to diagnose lymphomas. Although treatments for pulmonary MALT lymphoma include surgery, radiation, and chemotherapy, an initial treatment strategy has yet to be established in daily clinical medicine. Furthermore, because the prognosis of the disease is good, watchful waiting is often chosen [4, 5].

Low-grade B-cell lymphomas do not characteristically respond well to chemotherapy, and complete remission cannot be achieved in many cases. Even when complete remission is achieved, patients often relapse after several years. Therefore, a new drug that can maintain remission over a long period is desired [6]. Yttrium-90 ibritumomab tiuxetan is an anti-CD20 monoclonal antibody labeled with a *β*-emitting radionuclide that irradiates within lymphomas [7, 8]. In recent years, this novel agent has been highlighted as radioimmunotherapy for CD20-positive low-grade lymphomas. We herein reported an elderly patient with extensive consolidation, which was diagnosed as bulky pulmonary MALT lymphoma, who was treated with yttrium-90 ibritumomab tiuxetan.

## 2. Case Report

**Figure 1:**
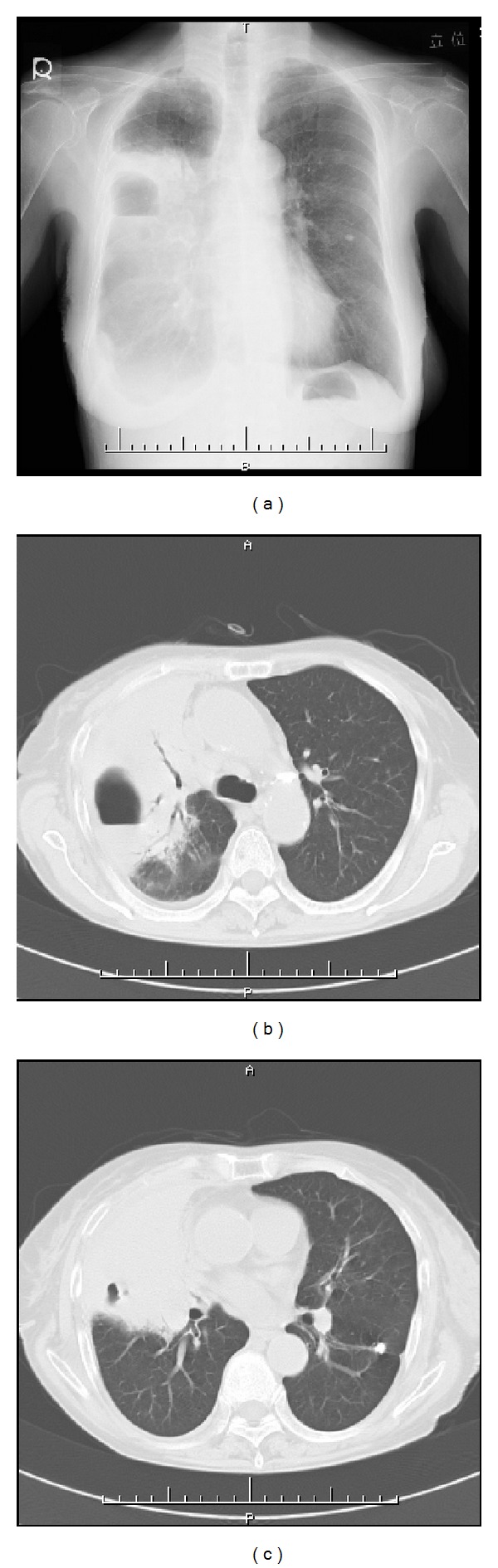
Chest X-ray (a) and CT ((b) and (c)) six years before the patient was referred to our hospital. CT revealed a lung abscess in the S2 field of the right lung and consolidation around this abscess.

An 84-year-old woman was admitted to a community hospital 6 years ago for a right-S2 lung abscess (Figure 1). She received antibiotic therapy and her clinical examinations improved after two weeks. Although chest CT scan on discharge revealed residual consolidations in the same field, no additional examinations were conducted. She underwent regular followups at the hospital for 6 years.

**Figure 2:**
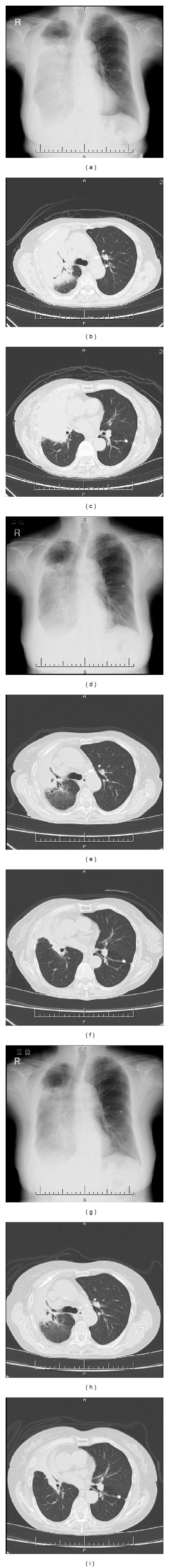
Chest X-ray (a) and CT scan ((b) and (c)) immediately before the administration of yttrium-90 ibritumomab tiuxetan. Chest X-ray (d) and CT ((e) and (f)) 2 months after its administration. Chest X-ray (g) and CT scan ((h) and (i)) 18 months after its administration. The abnormal shadow was significantly reduced after the administration of yttrium-90 ibritumomab tiuxetan. Radiation pneumonitis has not been reported for 18 months.

**Figure 3:**
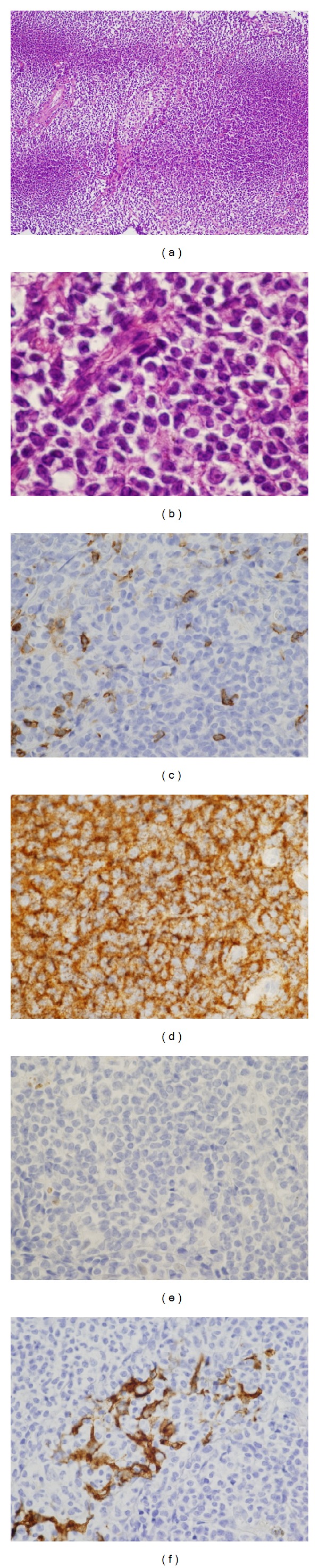
Microscopic findings of lung biopsy specimens with hematoxylin and eosin staining ((a) ×10; (b) ×80). Immunohistochemical examinations of the tissue were positive for CD20 ((d) ×40) and negative for CD5 ((c) ×40), bcl-1 ((e) ×40), and AE1/AE3 ((f) ×40).

She visited our hospital with nonproductive cough and dyspnea on exertion for one month (Medical Research Council dyspnea scale (MRC) grade 3). Chest X-rays revealed diffuse infiltrates in the middle and lower fields of the right lung (Figure 2(a)). CT scan showed consolidation with air bronchogram in the part of the right superior lobe that extended from the middle lobe to the inferior lobe (Figures 2(b) and 2(c)). Although the mediastinal lymph nodes were enlarged, the other lymph nodes were not swollen. From these results, we suspected bacterial pneumonia and primary lung cancer, and she was admitted to our hospital for further examinations. Laboratory results at that time revealed no abnormalities with a white blood cell count of 5,500/*μ*L, hemoglobin 13.4 g/dL, and platelet count 14.5 × 10^4^/*μ*L. However, LDH and soluble IL-2 receptor were high at 282 IU/L and 4,196 U/mL, respectively. These results required differentiation from malignant lymphoma. She did not have autoimmune disorders including Sjögren's syndrome by serological findings. Pulmonary function tests revealed restrictive impairment with a vital capacity (VC) of 65%. After hospitalization, we performed bronchoscopy with TBLB from the right B4. These lung specimens revealed no pathogens or malignant findings. CT-guided lung biopsy was then performed for consolidation in the right S4. Hematoxylin and eosin staining showed the diffuse infiltration of medium-sized lymphocytes in the tissue (Figures 3(a) and 3(b)). Lymphocytes with Dutcher bodies were observed in some parts. The majority of medium-sized lymphocytes were CD20 positive but were negative for CD5, bcl-1, and AE1/AE3 (Figures 3(c)–3(f)). Therefore, we diagnosed the patient with pulmonary MALT lymphoma. Her bone marrow included 0.8% abnormal lymphocytes that were hard to distinguish from lymphoma cells. In addition, fluorescence *in situ* hybridization (FISH) analysis on interphase nuclei revealed API2-MALT1 fusion signals in 11% of cells in the sample. These results supported the bone marrow infiltration of MALT lymphoma cells.

Since she exhibited bulky pulmonary MALT lymphoma with respiratory symptoms, we treated her with systemic chemotherapy. The patient initially received 4 cycles of rituximab weekly, as described by Conconi et al. [9]. We cytogenetically confirmed the absence of lymphoma cells in the bone marrow two months after chemotherapy. However, the abnormal shadow did not show any changes and she still exhibited respiratory symptoms. Therefore, we chose yttrium-90 ibritumomab tiuxetan, to which tolerance was expected from elderly patients with refractory or relapsed indolent non-Hodgkin's lymphoma [7, 8]. She was hospitalized, administered yttrium-90 ibritumomab tiuxetan, and was then discharged soon thereafter. Grade 4 thrombopenia was observed on day 27 and she was rehospitalized for a platelet transfusion. Grade 4 neutropenia was observed on day 32 and G-CSF was administered subcutaneously on a daily basis. However, no infection was observed during that period. Her bone marrow recovered on day 38, and she was safely discharged. As expected, she had no nonhematological toxicities or decrease in her performance status. CT scan performed on the second month following the administration of yttrium-90 ibritumomab tiuxetan revealed partial remission (Figures 2(d)–2(f)). Swelling was not also observed in the mediastinal lymph nodes. Her respiratory symptoms disappeared at that time (MRC grade 0) and %VC normalized to 82%. Eighteen months after the administration of yttrium-90 ibritumomab tiuxetan, CT scans showed no progression or radiation pneumonitis (Figures 2(g)–2(i)). Serum levels of LDH and soluble IL-2 receptor were also within normal ranges.

## 3. Discussion

CT imaging of pulmonary MALT lymphoma is capable of revealing various features such as tumor shadows, nodular shadows, consolidation, and ground-glass opacity shadows, which may be combined in many cases [4]. Few features are specific to pulmonary MALT lymphoma and it is difficult to distinguish lymphomas from bacterial pneumonia and lung cancer using only CT scans. Therefore, lung biopsy is essential for a definitive diagnosis. In our case, a diagnosis was made using CT-guided lung biopsy. The histopathological characteristics of MALT lymphoma have been shown to include lymphoepithelial lesions, plasma cell differentiation, and Dutcher bodies, and trisomy 3 and t(11;18)(q21;q21) are also characteristic of chromosomal abnormalities [10, 11]. The API2-MALT1 fusion gene occurs by the translocation of two genes, identified as the API2 gene at 11q21 and MALT1 gene at 18q21, and translocation is particularly high at approximately 50% in pulmonary MALT lymphoma [11]. Since the lung biopsy specimen obtained was very small, we could not identify the API2-MALT1 fusion gene. However, the API2-MALT1 fusion gene was cytogenetically identified in the bone marrow using the FISH analysis, which supported bone marrow infiltration, and was an important examination to determine the initial therapy.

The initial therapy of MALT lymphoma varies according to the originating organs. When localized, such as in gastric MALT lymphomas, *Helicobacter pylori* elimination is performed [12]. External irradiation is first chosen for thyroid or orbital MALT lymphomas, while treatment options for pulmonary MALT lymphomas also include watchful waiting due to radiation pneumonitis [4, 13, 14]. Furthermore, progression-free survival (PFS) for pulmonary MALT lymphoma has been shown to be poorer with combinating chemotherapy using cyclophosphamide and anthracycline than with chlorambucil alone, and standard chemotherapy has not yet been established [4]. Conconi et al. showed that 4 cycles of rituximab weekly for MALT lymphomas in various organs led to good outcomes with an overall response rate (ORR) of 73% [9]. On the other hand, the complete remission rate (CRR) was low and there was a high rate of recurrence. In the present case, we administered 4 cycles of rituximab weekly as initial therapy. We could not detect API2-MALT1 fusion genes in the bone marrow, which indicated that rituximab alone reduced lymphoma cells disseminating throughout the organ. However, no marked changes in her pulmonary images or respiratory symptoms were observed 2 months after the initial therapy. Thus, systemic chemotherapy without cyclophosphamide and anthracycline was required for further remission. Recently, it has been reported that rituximab plus chlorambucil in patients with MALT lymphoma improve better ORR and event-free survival compared to chlorambucil alone, with low toxicities [15]. In Japan, the chlorambucil has not been approved.

Malignant lymphomas generally respond to chemotherapy and are highly sensitive to radiation. Yttrium-90 ibritumomab tiuxetan, which was developed as radioimmunotherapy, has recently become available in routine clinical practice in Japan. A phase II trial on yttrium-90 ibritumomab tiuxetan in Japan resulted in an ORR of 83% for refractory or recurrent low-grade B-cell lymphoma, a CRR of 68%, and a mean PFS of 9.6 months, indicating good outcomes [16]. Esmaeli et al. treated 12 patients with extranodal indolent ocular adnexal lymphomas with yttrium-90 ibritumomab tiuxetan as a front-line strategy and obtained an ORR of 100% (CRR 83%) with no distal recurrence during the follow-up period [17]. The main adverse effects associated with this therapy were reported to be hematotoxicities, with G-CSF or transfusion therapy being necessary in approximately 20% of cases at approximately 6–8 weeks [7, 16]. The severity of hematotoxicities correlated well with bone marrow infiltration and postchemotherapy [7, 8, 16]. Most nonhematotoxicities have been shown to be mild and tolerable, which makes yttrium-90 ibritumomab tiuxetan administration possible even for elderly patients. Although grade 4 hematotoxicities were temporarily observed in our case, non-hematotoxicities were not reported, which was similar to the trials on yttrium-90 ibritumomab tiuxetan described above.

Similar to external radiation, radiation damage in various organs needs to be considered with *β*-particle emitting radioimmunotherapy [8]. No cases of cataracts, dry eye, or retinitis were reported in recent studies on yttrium-90 ibritumomab tiuxetan administered for extranodal indolent ocular adnexal lymphomas [17]. The estimated total radiation dose with the agent around the orbital cavity was shown to be approximately 3 Gy, which is nearly one tenth of the external radiation dose [18]. On the other hand, there have only been 2 reported cases worldwide of radiation-induced pulmonary fibrosis by yttrium-90 ibritumomab tiuxetan. One of these was a fatal case, indicating that radiation-induced pulmonary fibrosis is an adverse event that requires consideration even though it is very rare [19, 20]. Although our case of bulky pulmonary MALT lymphoma did not exhibit radiation pneumonitis, continued careful followup is needed.

## 4. Conclusions

Yttrium-90 ibritumomab tiuxetan is an effective treatment for MALT lymphoma originating from organs in which damage to normal tissue by external irradiation could not be avoided and for elderly people that could not tolerate the adverse effects associated with other chemotherapies. We here chose yttrium-90 ibritumomab tiuxetan as salvage therapy for bulky pulmonary MALT lymphoma and obtained a good outcome. We suggest that yttrium-90 ibritumomab tiuxetan may be included as a treatment option for pulmonary MALT lymphoma with respiratory symptoms.
